# Adaptive convergence at the genomic level—prevalent, uncommon or very rare?

**DOI:** 10.1093/nsr/nwaa076

**Published:** 2020-04-24

**Authors:** Ziwen He, Shaohua Xu, Suhua Shi

**Affiliations:** School of Life Sciences, Sun Yat-sen University, China; School of Life Sciences, Sun Yat-sen University, China; School of Life Sciences, Sun Yat-sen University, China

Convergent evolution is one of the central topics in evolutionary genetics [1]. While there has been ample evidence of phenotypic convergence, the issue is whether each and any of the phenotypic convergences have an underlying cause in genic convergence [2,3]. Fortunately, the torrent of genomic data has made it possible to address the issue [4–8]. Convergence can happen at multiple levels of the genetic architecture. For example, many studies have reached the conclusion of genic convergence when the same gene has experienced many more amino acid (AA) changes than expected [3,9–11]. Another prominent example is the TCGA (The Cancer Genome Atlas) data on the evolution of tumors, whereby convergence is defined as the sharing of mutations in the same genes, rather than the same change at the same site [12,13]. Many others have further relaxed the stringency in defining convergent evolution. For example, convergence could also mean copy number or evolutionary rate changes in the same genes [14,15].

In this perspective, we survey the literature on systems that fulfill the most stringent criterion for convergent molecular evolution—namely, the same sites of the same gene have independently evolved to the same AA. This definition of molecular convergence has been more commonly adopted in the literature [9,16,17] than the various other criteria with relaxed stringency, which have mostly been narrowly applied.

We wish to make two additional points about molecular convergence. First, the convergence literature sometimes makes a distinction between convergent evolution and parallel evolution. The former term applies when two species evolve from different ancestral states (A and B) to the same new state C whereas the latter refers to the evolution from the same state A to the same new state C. This distinction cannot be applied to the molecular data because the independent evolution A → C and B → C is so common that de-noising would be virtually impossible. Hence, molecular convergence in this study means the independent evolution from the same old state to the same new state. Second, we refer to adaptive convergence simply as ‘convergence’ while chance convergence is referred to as background convergence (or simply ‘noise’).

By the above definition of AA-site convergence, there are two classes of convergence studies, to be referred to as the genic and the genomic approaches, respectively. In the genic approach, a set of genes has been pre-determined based on prior knowledge of the phenotypes (e.g. lactase persistence [18]). Supplementary Table 1 shows 35 such cases. With the information on the branch lengths and amino acid substitution patterns, the expected level of background convergence can be calculated [9,19]. Because of the small number of genes involved, the probability that any of them will show signs of molecular convergence by chance is generally quite small; hence, the use of a control group for comparison is often unnecessary. By and large, studies taking the genic approach are uncontroversial.

The genic approach is limited by the known genetic mechanisms underlying the phenotypes of interest. The availability of whole genomic data has the potential to break the limits when the genetic basis of the phenotype is not known. Nevertheless, the statistics of inferring convergence by the genomic approach is far more challenging because the large genomes are liable to incur extensive background convergence (i.e. noises). In Table 1, we list 14 studies that report convergence at the genomic level. Most published studies on genomic convergence take a theoretical approach to estimating the amount of background convergence (i.e. the noise level). For example, if three taxa have independently invaded a new habitat (say desert; see Fig. 1), the simulations may show that, by chance alone, 1000 AA substitutions in the entire genomes would be shared by the desert taxa. Furthermore, few of these 1000 substitutions are found in the three non-desert control taxa. If the observed AA substitutions shared by desert taxa are 1500 in number, then it is concluded that 500 AA substitutions occur by convergence.

The major deficiency of such theoretical calculations is the absence of validation as investigators may often under-estimate the noise level. In the hypothetical example above, there might be 1600, instead of 1000 background AA sites, by chance alone if one uses parameter values that do not stay constant. Any variation in nature that is not factored into the calculation would lead to an under-estimation of the noise level. For example, by ignoring the variation of acceptable amino acids among different sites and at different genetic distances, one is likely to underestimate nonadaptive convergence [19,20].

**Table 1.** Publications on genomic convergence in and before 2019.

Species	Kingdom	Phenotype	Detecting method	No. of convergent genes	Reference
Echolocating bats and whales	Animalia	Echolocation	ΔSSLS (phylogenetical clustering)	∼200	Parker *et al*. [4]
Echolocating bats and whales	Animalia	Echolocation	Convergent substitution counting	392	Lee *et al.* [30]
Marine mammals (whale, walrus, manatee)	Animalia	Adaptation to marine environment	Counting + Positive selection (PAML)	8	Foote *et al*. [5]
Giant and red pandas	Animalia	Bamboo diet, pseudothumb	Exceeding theoretical estimation + Positive selection (PAML)	70	Hu *et al*. [11]
Flight degeneration birds	Animalia	Loss of flight	Divergence + Association mapping	2	Pan *et al*. [31]
Yak and Tibetan antelope	Animalia	Adaptation to high altitude	Exceeding theoretical estimation + Positive selection (PAML)	1	Wang *et al*. [10]
Pseudomonas aeruginosa (intraspecies)	Animalia	Host adaptation	Exceeding theoretical estimation	52	Marvig *et al*. [32]
Crassulacean acid metabolism (CAM) species	Plantae	Crassulacean acid metabolism (CAM)	Counting + Phylogenetical clustering	4	Yang *et al*. [33]
Extremophile fishes (ecotypes within species)	Animalia	Adaptation to hydrogen sulfide (H2S)-rich environments	Phylogenetical clustering	∼1.2% of genomic window	Brown *et al*. [34]
Lake and stream stickleback	Animalia	Adaptation to lake or stream environment	Divergence + Association mapping	∼2% of genomic window	Rennison *et al*. [35]
*Arabidopsis halleri* and *A. arenosa*	Plantae	Adaptation to calamine metalliferous soils	Divergence + Association mapping	24	Preite *et al*. [36]
Stony corals	Animalia	Symbiont transmission mode	Convergent substitution counting	403	Dixon and Kenkel [37]
Plateau zokor and naked mole rat	Animalia	Subterranean environments	Convergent substitution counting	787	Shao *et al.* [38]
Lodgepole pine and interior spruce	Plantae	Spatial variation in temperature	Association mapping	47	Yeaman *et al.* [39]

By this reasoning, types of ‘empirical control’ are needed. A simplest form of the control is the observed convergence among the three non-desert taxa. If the ‘empirical control’ also yields 1500 convergent AAs, then it would be difficult to conclude any true convergent sites among the desert taxa. An important feature of the phylogeny of Fig. 1 is the symmetry between the desert and non-desert taxa—each focus species is paired with

a control species (see the CCS (convergence at conservative sites) method below). We further note that all statistical treatments applied to the focus group should be applied equally to the control taxa. For example, studies often filter the genes by algorithms that choose positively selected genes for analysis as in studies of pandas, marine mammals and Tibetan animals [5,10,11]. When such a procedure is used on the focus group, it should be used on the empirical control as well, a practice rarely adopted.

Curiously, only two of the 14 genomic studies in Table 1 have been tested by an ‘empirical control’. In both cases, the theoretical inferences are nullified by the empirical control. These two studies [4,5], together with the follow-up analyses [6–8], underlie the main

argument of this perspective: because of the high likelihood of under-estimating the background noises in theoretical models, the empirical control is indispensable for site convergence studies.

Parker *et al*. [4] studied mammals with echolocation capabilities (bats and whales) and identified 200 genes of adaptive convergence, using a method referred to as ΔSSLS. The empirical controls were done by two subsequent studies. Both Zou and Zhang [7] and Thomas and Hahn [6] found that the level of convergence between non-echolocating mammals (or between an echolocating and a non-echolocating species) is the same as between echolocating mammals. This may be the very first indication of the importance of the empirical control. When applied solely to the focus group (i.e. echolocating mammals), the ΔSSLS method yields mainly false positives. This is likely true for other one-sided analyses that only examine the focus group without an empirical control [5,10,11].

**Figure 1.**
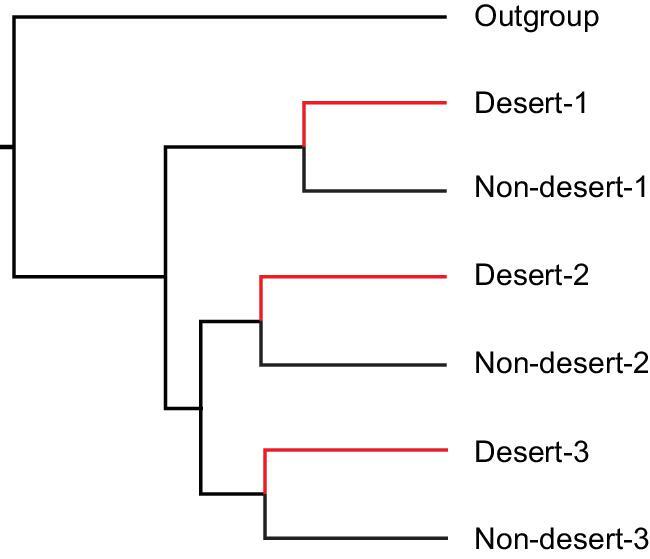
A symmetric design for detecting convergence among three hypothetical taxa that colonize the desert habitat independently. Each focus desert species is paired with a non-desert control. In such a design, the three non-desert species provide the empirical control without requiring parameter inputs in determining the background level of convergence. In the text, this design is proposed to be a key step in any genomic analysis of convergence.

In the second study, Foote *et al.* [5] analyzed 15 mammalian genomes for convergent signals in three marine taxa—killer whale/dolphin, walrus and manatee. This may be the only genomic study that incorporates an empirical control. Foote *et al.* listed specific adaptive amino acid substitutions that have evolved by convergence but, at the same time, reported ‘higher levels of convergent amino acid substitutions in a control set of terrestrial sister taxa to the marine mammals’. In other words, background noises appear to overwhelm the signals of convergence. Foote *et al*. made an interesting argument in this context: although the noise level is high, it does not necessarily mean that the detected signals are false. (In particular, the signals have been selected by using the Phylogenetic Analysis by Maximum Likelihood (PAML) algorithm [21] to identify adaptive genes but it is not clear why the same treatment was not used on the control.)

Following this logic, a method for detecting convergence by stringently filtering out noises should be most useful. The CCS method proposed by Xu *et al*. [8] seems a snug fit for this purpose. The CCS method first builds a symmetric phylogeny between the focal and control group, then detects convergence at the conservative sites in both groups. The CCS method has a three-fold advantage in detecting true adaptive convergence [8]. First, conservatively evolving sites are less afflicted by random noises. Second, conservative genes undergoing accelerated evolution in a new environment often harbor candidate sites of adaptation [15,22] and convergent evolution is built on such newly adaptive changes. Third, because CCS is a symmetric design as shown in Fig. 1, the convergence level in the control group can be used directly on the focus group as the background noise. (An additional concern about unequal evolutionary rates among branches can be found in Xu *et al.* [8].)

Xu *et al*. apply the CCS method to mangrove trees and report that, even after reducing background noises to the possible minimum, one could only infer the convergence with 50% certainty [8]. Thus, convergence signals are probabilistic in nature and convergent sites could not be unambiguously pinpointed. Although CCS method might risk high false negatives, eliminating false positives should be the goal at present as false positives are still overwhelmingly prevalent. The CCS method has recently been updated to use a non-symmetric design that substantially increases the power of detecting true convergence [23].

We now re-analyze the 13 mammalian genomes (Fig. 2), following the set-up of Foote *et al.* [5]. This is also the symmetric design required by Xu *et al.* [8]. A total of 16 873 orthologous alignments were provided by Foote *et al.* [5]. Using the CCS method, we first define conservative sites and then identify convergent amino acid substitutions among them. The conservative sites are identified if the three inland controls have the same amino acid state ‘O’ (N_1_ = N_2_ = N_3_ = O) and the other seven land mammals have either ‘O’ or missing data. When so defined, the ancestral state can be confidently inferred to be ‘O’, as described in Xu *et al.* [8]. Given the ancestral state of O, convergence can be defined if two or more of the marine mammals share the same derived state (M_i_ = M_j_ ≠ O). In contrast, divergent sites are those with M_i_ ≠ O, M_j_ ≠ O and M_i_ ≠ M_j_. The same criteria, with marine mammals and their inland relatives switched, are applied to infer convergence among the control taxa (Fig. 2).

**Figure 2.**
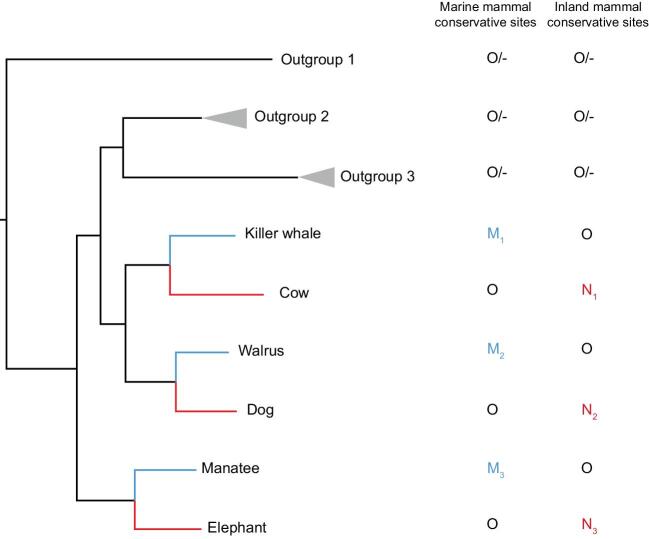
Mammalian species used in the convergence test. O indicates the character state of the Outgroup 1 (Opossum), Outgroup 2 (Human, Rhesus macaque, Baboon and Marmoset) and Outgroup 3 (Mouse and Rat). M_i_ and N_i_ indicate the character state in marine (blue) and inland mammals (red), respectively. In the CCS method, convergence is inferred only at conservative sites where N_1_ = N_2_ = N_3_ = O and the other seven inland mammals have either ‘O’ or ‘-’ (missing data, no more than five species). Convergence is inferred when M_i_ = M_j_ ≠ O. For the control, the same criteria, with M_i_ and N_i_ switched, are applied. The phylogenetic tree is reconstructed using 1000 randomly picked protein alignments.

**Table 2.** Convergent and divergent sites detected by the CCS method. Only conservative sites are used in the CCS method. T, C and D represent total, convergent and divergent site numbers. (The description of divergent sites is given in the Supplementary Data.)

	Total conservative sites (T)	Divergent sites (D)	Convergent sites (C)	C/T	C/D	Number of genes with n convergent sites (n = 1, 2, 3)
Marine mammals	2 302 244	1558	1282	5.6 × 10^−4^	0.823	930: 135: 24 (85%: 12%: 2%)
Inland mammals	2 977 271	2502	1861	6.3 × 10^−4^	0.744	1198: 204: 74 (81%: 14%: 5%)

From the analysis presented in Table 2, 1282 and 1861 convergent substitutions are detected in the marine and inland mammals, respectively. As expected, the proportions of conservative sites that show convergent substitutions (the C/T ratio in Table 2) are low, at 5.6 × 10^−4^ and 6.3 × 10^−4^. Therefore, when the background noises are reduced to the minimum, the marine mammals still show a lower signal of genome convergence than the inland relatives. The convergence detected is apparently the residual noises that could not be further purged. In the Supplementary Data, we present a more detailed analysis, which confirms that, overall, marine mammals do not yield site-convergence signals.

In detecting molecular convergence, the choice of taxa from similar, if not identical, environments should be the most important. In the case of marine mammals, walruses are found in the Arctic while manatees exist in tropical waters [24,25]. Killer whales and dolphins range more widely in both cold and warm waters than the other two taxa [26]. Furthermore, walruses only come into water for feeding whereas whales and manatees are obligatorily aquatic. It does not seem compelling that such dissimilar

selective pressures would lead tothe same molecular outcome. In such taxa, weak genomic convergence may not be surprising. Ideal candidates for genomic convergence detection should be taxa adapted to the same environment (or highly similar ones) for nearly the same amount of time. The candidates may include the large collection of woody plants that invade the tropical coasts, known as mangroves, at a comparable time [27–29].

In the search for convergence signals, the best way to estimate the background noises would not be by simulations or theoretical calculations. Given the vicissitude of sequence evolution, we recommend the use of empirical controls that are symmetrically placed. Among the 14 genomic studies of convergence, only two have such controls, both of which yield a level of background convergence that is the same or higher than that of the focus taxa. It is prudent to suggest that, at the genomic level, there is so far no evidence of convergence. In the future, it will be necessary to start the search using a symmetric model (e.g. CCS) that can yield a set of candidate genes with a stronger signal than noise. Further analysis to identify the genes of true convergence can then be extended from this basic design.

Finally, all evidence based on sequence comparisons can still, in principle, be false positives. Hence, the clinching proof will have to be functional tests. At its most basic level, genomic analysis is to identify candidate genes, on which functional tests will be able to provide the proof of convergent adaptation.

## Supplementary Material

nwaa076_Supplemental_File
