# Supplementary material for: Adaptive convergence at the genomic level—prevalent, uncommon or very rare?
Source: Natl Sci Rev. 2020 Apr 24;7(6):947–51. doi: 10.1093/nsr/nwaa076 (PMC8289048; doi:10.1093/nsr/nwaa076)
Supplement: nwaa076_Supplemental_File [file nwaa076_supplemental_file.pdf]

Supplementary Information of

Adaptive convergence at the genomic level – Prevalent, uncommon or very rare?

Supplementary text

Since there is no signal above the residual noise, we ask what may be the upper bound by a less stringent test. The C/D ratio is such a test (see Table 2) where C is the number of convergent substitutions and D is the number of divergent substitutions. Both convergent and divergent sites are identified from the conservative sites. The C/D ratio in marine mammals is 0.823 (1282/1558) and in inland mammals is 0.744 (1861/2502). While there may be proportionately more convergent sites in marine mammals, the pattern may also mean that the inland mammals have more divergent sites. We suspect that, in the inland mammals, there might be more divergent substitutions, which are indeed 61% higher (2502/1558) than that in the marine mammals.

Even if we assume that the C/D ratio difference is due solely to the stronger convergence in marine mammals (rather than the larger divergence in the control), we can obtain an upper bound of the estimate in the marine mammals. In this case, 123 (1282 - 1558 × 0.744) of the 1282 convergent sites are candidates for adaptive convergence. At the risk of over-estimating convergence by using the C/D test, each of the 1282 convergent sites in marine mammals would have a < 10% (0.823 - 0.744) chance of being the true signal.

If the same selective pressure operates on certain genes in marine mammals (e.g. genes governing the development of fur or breathing physiology), one might expect such genes to have disproportionately more convergent substitutions. The last column of Table 2 does not support this supposition. In fact, marine mammals have proportionately fewer genes with > 1 convergent sites than their inland relatives. Overall, marine mammals do not yield convincing site-convergence signals.

Supplementary Table 1. Selected examples of convergent genetic evolution. Note that the table includes five cases of experimental evolution in bacteria or fungi genomes. Considering the small number of genes in these unicellular organisms, we treat these cases as genic instead of genomic convergence. The cases masked by asterisk have been summarized in Stern 2013 [16].

Species	Kingdom	Taxonomic level	Phenotype	Genes	References
Escherichia coli	Monera	Intraspecific (experimental evolution)	Adaptation to glucose-limited medium	Multiple genes *	Woods *et al*. [39]
		Intraspecific (experimental evolution)	Adaptation to glycerol-based medium	Glycerol kinase (*glpK*) and RNA polymerase genes *	Herring *et al*. [40]
Pseudomonas aeruginosa	Monera	Intraspecific (experimental evolution)	Adaptation to novel environments	Multiple genes *	Wong *et al*. [41]
		Intraspecific (experimental evolution)	Hyperswarming	Flagella synthesis regulator (*fleN*) *	van Ditmarsch *et al*. [42]
Saccharomyces cerevisiae	Fungi	Intraspecific (experimental evolution)	Adaptation to fluctuating glucose and galactose levels	*GAL80* *	Segre *et al*. [43]
Diverse species of yeast	Fungi	Interspecific	Loss of galactose utilization	*GAL* genes *	Hittinger *et al*. [44]
Ipomoea horsfalliae and *Ipomoea quamoclit*	Plantae	Intergeneric	Evolution of red flowers from blue flowers	Flavonoid 3′-hydroxylase *	Streisfeld & Rausher [45]

Arabidopsis thaliana and *Arabidopsis lyrata*	Plantae	Intraspecific and interspecific	Vernalization	*FRIGIDA* *	Johanson *et al.*; Kuittinen *et al.* [46,47]
Plants (multiple species)	Plantae	Interspecific	C4 photosynthesis	Phosphoenolpyruvate carboxylases (PEPC) genes *	Christin *et al.* 2007; Blasing *et al.* 2000; Besnard *et al.* 2009 [48–50]
Human (*Homo sapiens*)	Animalia	Intraspecific	Resistance to malaria	Glucose-6-phosphate dehydrogenase (*G6PD*) *	Tishkoff *et al.* 2001 [51]
		Intraspecific	Lactase persistence	Lactase (*LCT*) *	Tishkoff *et al.* 2007 [18]
		Intraspecific	Skin lightening	*OCA2*	Yang *et al.* 2016 [52]
Colobine leaf-eating monkeys (*Pygathrix nemaeus* and *Colobus guereza*)	Animalia	Interspecific	Enhanced digestive efficiency	RNase gene *	Zhang 2006 [2]
Cave fish (*Astyanax mexicanus*)	Animalia	Intraspecific	Albinism	Oculocutaneous albinism II (*Oca2*)	Protas *et al.* 2006 [53]
		Intraspecific	Reduced pigmentation	Melanocortin 1 receptor (*Mc1r*) *	Gross *et al.* 2009 [54]
Cichlid species from Lake Tanganyika and Lake Malawi	Animalia	Interspecific	Spectral sensitivity	Rhodopsin gene *	Sugawara *et al.* 2005 [55]
Pufferfish (*Takifugu rubripes* and *Tetraodon nigroviridis*) and clam (*Mya arenaria*)	Animalia	Interspecific	Tetrodotoxin resistance	Sodium channel gene *	Bricelj *et al.* 2005; Venkatesh *et al.* 2005 [56,57]

Drosophila spp.	Animalia	Interspecific	Trichome patterning	*shavenbaby* *	Sucena & Stern 2000; Sucena *et al*. 2003; Frankel *et al*. 2012 [58–60]
Insects (multiple species)	Animalia	Interspecific	Cardenolide resistance	(Na++K+) ATPase gene *	Dobler *et al*. 2012; Zhen *et al*. 2012 [61,62]
Insects, amphibins, reptiles, mammals	Animalia	Interspecific	Resistance to toxic cardiac glycosides	(Na++K+) ATPase gene	Ujvari *et al*. 2015 [63]
Stickleback (*Gasterosteus aculeatus*)	Animalia	Intraspecific	Pelvic spine and girdle reduction	Paired-like homeodomain transcription factor 1 (*Pitx1*) *	Chan *et al*. 2010; Shapiro *et al*. 2004; Cresko *et al*. 2004 [64–66]
Sticklebacks (multiple species)	Animalia	Interspecific	Lateral plates	*Ectodysplasin* *	Colosimo *et al*. 2005; Cresko *et al*. 2004 [66,67]
Hummingbirds	Animalia	Interspecific	high-altitude adaptation	*Hemoglobin*	Projecto-Garcia *et al*. 2013 [68]
Waterfowl (Birds)	Animalia		High-altitude adaptation	*hemoglobin*	Natarajan *et al*. 2015 [69]
Snakes, newts, puffer fish	Animalia	Interspecific	Resistance to TTX (tetrodotoxin)	voltage-gated sodium channels (Na_V1)	Feldman *et al*. 2012; Brodie and Brodie 2015 [70,71]
Echolocating mammals (bats & toothed whales)	Animalia	Interspecific	Echolocation	*prestin*, *KCNQ4*, *Cdh23*, *Pcdh15*, *Otof*	Liu *et al*. 2010; Li *et al*. 2010; Liu *et al*. 2012; Shen *et al*. 2012; Liu *et al*. 2018 [3,72–75]
High-elevation amphibian species	Animalia	Interspecific	High-altitude adaptation	*MYBPC2*	Yang *et al*. 2017 [76]

Agamid lizards & snakes	Animalia	Interspecific	Metabolic function	13 protein-coding mitochondrial gene	Castoe *et al.* 2009 [77]
Cetaceans & Carnivores	Animalia	Interspecific	Carnivorous	*CTRC*, *CYP7A1*, *PNLIP*	Wang *et al.* 2016 [78]
Hypoxia-tolerant mammals	Animalia	Interspecific	Hypoxia adaptation	*HIF1A*, *HIF2A*, *HIF1B*, *PHD3*	Zhu *et al.* 2018 [79]
high-elevation anurans	Animalia	Interspecific	High-altitude adaptation	*HSP90AA1*	Jin *et al.* 2018 [80]
Mosquito	Animalia	Intraspecific and interspecific	Insecticide resistance	Acetylcholinesterase (Ace-1)	Weill *et al.* 2003; Weill *et al.* 2004; Alout *et al.* 2007 [81–83]
Musca domestica, Lucilia cuprina & Lucilia sericata	Animalia	Interspecific	Organophosphate insecticide resistance	esterase isozyme E3	Hartley *et al.* 2006; Claudianos *et al.* 1999 [84,85]
Bird (Hoztzin), cattle & colobine monkey	Animalia	Interspecific	Anaerobic digestion	lysozyme	Swanson *et al.* 1991; Kornegay *et al.* 1994; Stewart *et al.* 1987; Messier and Stewart 1997 [86–89]
Bananaquit & chicken	Animalia	Interspecific	Pigmentation (feathers)	*MC1R*	Theron *et al.* 2001; Takeuchi *et al.* 1996; Kerje *et al.* 2003 [90–92]

Supplementary References

39. Woods R, Schneider D, Winkworth CL *et al.* Tests of parallel molecular evolution in a long-term experiment with Escherichia coli. *Proc Natl Acad Sci U S A* 2006;**103**:9107–12.

40. Herring CD, Raghunathan A, Honisch C *et al.* Comparative genome sequencing of Escherichia coli allows observation of bacterial evolution on a laboratory timescale. *Nat Genet* 2006;**38**:1406–12.

41. Wong A, Rodrigue N, Kassen R. Genomics of Adaptation during Experimental Evolution of the Opportunistic Pathogen Pseudomonas aeruginosa. *PLoS Genet* 2012;**8**, DOI: 10.1371/journal.pgen.1002928.

42. van Ditmarsch D, Boyle KE, Sakhtah H *et al.* Convergent evolution of hyperswarming leads to impaired biofilm formation in pathogenic bacteria. *Cell Rep* 2013;**4**:697–708.

43. Segrè A V, Murray AW, Leu J-Y. High-Resolution Mutation Mapping Reveals Parallel Experimental Evolution in Yeast. *PLOS Biol* 2006;**4**:e256.

44. Hittinger CT, Rokas A, Carroll SB. Parallel inactivation of multiple GAL pathway genes and ecological diversification in yeasts. *Proc Natl Acad Sci U S A* 2004;**101**:14144–9.

45. Streisfeld MA, Rausher MD. Genetic changes contributing to the parallel evolution of red floral pigmentation among Ipomoea species. *New Phytol* 2009;**183**:751–63.

46. Johanson U, West J, Lister C *et al.* Molecular analysis of FRIGIDA, a major determinant of natural variation in Arabidopsis flowering time. *Science* 2000;**290**:344–7.

47. Kuittinen H, Niittyvuopio A, Rinne P *et al.* Natural variation in Arabidopsis lyrata vernalization requirement conferred by a FRIGIDA indel polymorphism. *Mol Biol Evol* 2008;**25**:319–29.

48. Christin PA, Salamin N, Savolainen V *et al.* C4 Photosynthesis Evolved in Grasses via Parallel Adaptive Genetic Changes. *Curr Biol* 2007;**17**:1241–7.

49. Bläsing OE, Westhoff P, Svensson P. Evolution of C4 phosphoenolpyruvate carboxylase in Flaveria, a conserved serine residue in the carboxyl-terminal part of the enzyme is a major determinant for C4-specific characteristics. *J Biol Chem* 2000;**275**:27917–23.

50. Besnard G, Muasya AM, Russier F *et al.* Phylogenomics of C4photosynthesis in sedges (Cyperaceae): Multiple appearances and genetic convergence. *Mol Biol Evol* 2009;**26**:1909–19.

51. Tishkoff SA, Varkonyi R, Cahinhinan N *et al.* Haplotype diversity and linkage disequilibrium at human G6PD: Recent origin of alleles that confer malarial resistance. *Science* 2001;**293**:455–62.

52. Yang Z, Zhong H, Chen J *et al.* A Genetic Mechanism for Convergent Skin Lightening during Recent Human Evolution. *Mol Biol Evol* 2016;**33**:1177–87.

53. Protas ME, Hersey C, Kochanek D *et al.* Genetic analysis of cavefish reveals molecular convergence in the evolution of albinism. *Nat Genet* 2006;**38**:107–11.

54. Gross JB, Borowsky R, Tabin CJ. A novel role for Mc1r in the parallel evolution of depigmentation in independent populations of the cavefish Astyanax mexicanus. *PLoS Genet* 2009;**5**, DOI: 10.1371/journal.pgen.1000326.

55. Sugawara T, Terai Y, Imai H *et al.* Parallelism of amino acid changes at the RH1 affecting spectral sensitivity among deep-water cichlids from Lakes Tanganyika and Malawi. *Proc Natl Acad Sci U S A* 2005;**102**:5448–53.

56. Bricelj VM, Connell L, Konoki K *et al.* Sodium channel mutation leading to saxitoxin resistance in clams increases risk of PSP. *Nature* 2005;**434**:763–7.

57. Venkatesh B, Lu SQ, Dandona N *et al.* Genetic basis of tetrodotoxin resistance in pufferfishes. *Curr Biol* 2005;**15**:2069–72.

58. Sucena É, Stern DL. Divergence of larval morphology between Drosophila sechellia and its sibling species caused by cis-regulatory evolution of ovo/shaven-baby. *Proc Natl Acad Sci U S A* 2000;**97**:4530–4.

59. Sucena E, Delon I, Jones I *et al.* Regulatory evolution of shavenbaby/ovo underlies multiple cases of morphological parallelism. *Nature* 2003;**424**:935–8.

60. Frankel N, Wang S, Stern DL. Conserved regulatory architecture underlies parallel genetic changes and convergent phenotypic evolution. *Proc Natl Acad Sci U S A* 2012;**109**:20975–9.

61. Dobler S, Dalla S, Wagschal V *et al.* Community-wide convergent evolution in insect adaptation to toxic cardenolides by substitutions in the Na,K-ATPase. *Proc Natl Acad Sci U S A* 2012;**109**:13040–5.

62. Zhen Y, Aardema ML, Medina EM *et al.* Parallel molecular evolution in an herbivore community. *Science* 2012;**337**:1634–7.

63. Ujvari B, Casewell NR, Sunagar K *et al.* Widespread convergence in toxin resistance by predictable molecular evolution. *Proc Natl Acad Sci U S A* 2015;**112**:11911–6.

64. Chan YF, Marks ME, Jones FC *et al.* Adaptive evolution of pelvic reduction in sticklebacks by recurrent deletion of a pitxl enhancer. *Science* 2010;**327**:302–5.

65. Shapiro MD, Marks ME, Peichel CL *et al.* Genetic and developmental basis of evolutionary pelvic reduction in threespine sticklebacks. *Nature* 2004;**428**:717–23.

66. Cresko WA, Amores A, Wilson C *et al.* Parallel genetic basis for repeated evolution of armor loss in Alaskan threespine stickleback populations. *Proc Natl Acad Sci U S A* 2004;**101**:6050–5.

67. Colosimo PF, Hosemann KE, Balabhadra S *et al.* Widespread parallel evolution in sticklebacks by repeated fixation of ectodysplasin alleles. *Science* 2005;**307**:1928–33.

68. Projecto-Garcia J, Natarajan C, Moriyama H *et al.* Repeated elevational transitions in hemoglobin function during the evolution of Andean hummingbirds. *Proc Natl Acad Sci* 2013;**110**:20669–74.

69. Natarajan C, Projecto-Garcia J, Moriyama H *et al.* Convergent Evolution of Hemoglobin Function in High-Altitude Andean Waterfowl Involves Limited Parallelism at the Molecular Sequence Level. *PLoS Genet* 2015;**11**:1–25.

70. Feldman CR, Brodie ED, Brodie ED *et al.* Constraint shapes convergence in tetrodotoxinresistant sodium channels of snakes. *Proc Natl Acad Sci U S A* 2012;**109**:4556–61.

71. Brodie ED, Brodie ED. Predictably convergent evolution of sodium channels in the arms race between predators and prey. *Brain Behav Evol* 2015;**86**:48–57.

72. Liu Y, Cotton JA, Shen B *et al.* Convergent sequence evolution between echolocating bats and dolphins. *Curr Biol* 2010;**20**:53–4.

73. Liu Y, Han N, Franchini LF *et al.* The voltage-gated potassium channel subfamily KQT member 4 (KCNQ4) displays parallel evolution in echolocating bats. *Mol Biol Evol* 2012;**29**:1441–50.

74. Shen YY, Liang L, Li GS *et al.* Parallel evolution of auditory genes for echolocation in bats and toothed whales. *PLoS Genet* 2012;**8**, DOI: 10.1371/journal.pgen.1002788.

75. Liu Z, Qi FY, Xu DM *et al.* Genomic and functional evidence reveals molecular insights into the origin of echolocation in whales. *Sci Adv* 2018;**4**:eaat8821.

76. Yang W, Lu B, Fu J. Molecular convergent evolution of the MYBPC2 gene among three high-elevation amphibian species. *J Mol Evol* 2017;**84**:139–43.

77. Castoe TA, de Koning APJ, Kim H-M *et al.* Evidence for an ancient adaptive episode of convergent molecular evolution. *Proc Natl Acad Sci U S A* 2009;**106**:8986–91.

78. Wang Z, Xu S, Du K *et al.* Evolution of digestive enzymes and RNASE1 provides insights into dietary switch of cetaceans. *Mol Biol Evol* 2016;**33**:3144–57.

79. Zhu K, Ge D, Wen Z *et al.* Evolutionary genetics of hypoxia and cold tolerance in mammals. *J Mol Evol* 2018;**86**:618–34.

80. Jin H, Lu B, Fu J. Massive molecular parallel evolution of the hsp90aa1 gene between high-elevation anurans. *Asian Herpetol Res* 2018;**9**:195–200.

81. Weill M, Luffalla G, Mogensen K *et al.* Insecticide resistance in mosquito vectors. *Nature* 2003;**423**:136–7.

82. Weill M, Malcolm C, Chandre F *et al.* The unique mutation in ace-1 giving high insecticide resistance is easily detectable in mosquito vectors. *Insect Mol Biol* 2004;**13**:1–7.

83. Alout H, Berthomieu A, Cui F *et al.* Different amino-acid substitutions confer insecticide resistance through acetylcholinesterase 1 insensitivity in Culex vishnui and Culex tritaeniorhynchus (Diptera: Culicidae) from China. *J Med Entomol* 2007;**44**:463–9.

84. Hartley CJ, Newcomb RD, Russell RJ *et al.* Amplification of DNA from preserved specimens shows blowflies were preadapted for the rapid evolution of insecticide resistance. *Proc Natl Acad Sci U S A* 2006;**103**:8757–62.

85. Claudianos C, Russell RJ, Oakeshott JG. The same amino acid substitution in orthologous esterases confers organophosphate resistance on the house fly and a blowfly. *Insect Biochem Mol Biol* 1999;**29**:675–86.

86. Swanson KW, Irwin DM, Wilson AC. Stomach lysozyme gene of the langur monkey: Tests for convergence and positive selection. *J Mol Evol* 1991;**33**:418–25.

87. Stewart CB, Schilling JW, Wilson AC. Adaptive evolution in the stomach lysozymes of foregut fermenters. *Nature* 1987;**330**:401–4.

88. Messier W, Stewart CB. Episodic adaptive evolution of primate lysozymes. *Nature* 1997;**385**:151–4.

89. Kornegay J, Schilling J, Wilson A. Molecular adaptation of a leaf-eating bird: stomach lysozyme of the hoatzin. *Mol Biol Evol* 1994;**11**:921–8.

90. Theron E, Hawkins K, Bermingham E *et al.* The molecular basis of an avian plumage polymorphism in the wild: A melanocortin-1-receptor point mutation is perfectly associated with the melanic plumage morph of the bananaquit, Coereba flaveola. *Curr Biol* 2001;**11**:550–7.

91. Kerje S, Lind J, Schütz K *et al.* Melanocortin 1-receptor (MC1R) mutations are associated with plumage colour in chicken. *Anim Genet* 2003;**34**:241–8.

92. Takeuchi S, Suzuki H, Yabuuchi M *et al.* A possible involvement of melanocortin 1-receptor in regulating feather color pigmentation in the chicken. *Biochim Biophys Acta - Gene Struct Expr* 1996;**1308**:164–8.
